# Balancing read length and sequencing depth: Optimizing Nanopore long‐read sequencing for monocots with an emphasis on the Liliales

**DOI:** 10.1002/aps3.11524

**Published:** 2023-06-06

**Authors:** Gisel Y. De La Cerda, Jacob B. Landis, Evan Eifler, Adriana I. Hernandez, Fay‐Wei Li, Jing Zhang, Carrie M. Tribble, Nisa Karimi, Patricia Chan, Thomas Givnish, Susan R. Strickler, Chelsea D. Specht

**Affiliations:** ^1^ School of Integrative Plant Science, Section of Plant Biology and the L. H. Bailey Hortorium Cornell University Ithaca New York 14853 USA; ^2^ BTI Computational Biology Center Boyce Thompson Institute Ithaca New York 14853 USA; ^3^ Department of Botany University of Wisconsin–Madison Madison Wisconsin 53706 USA; ^4^ School of Life Sciences University of Hawaiʻi, Mānoa Honolulu Hawaiʻi 96822 USA; ^5^ Present address: Plant Science and Conservation Chicago Botanic Garden Glencoe Illinois 60022 USA; ^6^ Present address: Plant Biology and Conservation Program Northwestern University Evanston Illinois 60208 USA

**Keywords:** *Calochortus*, genome sequencing, Liliaceae, MinION, N50, Oxford Nanopore, read length

## Abstract

**Premise:**

We present approaches used to generate long‐read Nanopore sequencing reads for the Liliales and demonstrate how modifications to standard protocols directly impact read length and total output. The goal is to help those interested in generating long‐read sequencing data determine which steps may be necessary for optimizing output and results.

**Methods:**

Four species of *Calochortus* (Liliaceae) were sequenced. Modifications made to sodium dodecyl sulfate (SDS) extractions and cleanup protocols included grinding with a mortar and pestle, using cut or wide‐bore tips, chloroform cleaning, bead cleaning, eliminating short fragments, and using highly purified DNA.

**Results:**

Steps taken to maximize read length can decrease overall output. Notably, the number of pores in a flow cell is correlated with the overall output, yet we did not see an association between the pore number and the read length or the number of reads produced.

**Discussion:**

Many factors contribute to the overall success of a Nanopore sequencing run. We showed the direct impact that several modifications to the DNA extraction and cleaning steps have on the total sequencing output, read size, and number of reads generated. We show a tradeoff between read length and the number of reads and, to a lesser extent, the total sequencing output, all of which are important factors for successful de novo genome assembly.

The field of plant genomics has expanded rapidly in the past 20 years, with recent genome assemblies being more contiguous and accurate than earlier assemblies largely due to the incorporation of long‐read sequencing technology (Marks et al., 2021). Having a representative assembled genome for any organismal system facilitates the identification of variants across individuals (both single nucleotide polymorphisms and structural variants); the characterization of gene family expansion or contraction; and the determination of genes under selection, population‐level genetic diversity, current and historic effective population sizes, and other factors associated with adaptation that are important for evolutionary, ecological, and biodiversity studies (Worley et al., 2017; Paez et al., 2022). While a chromosome‐scale assembly is often desired, a draft genome assembly is often sufficient for exploring most evolutionary questions. A draft assembly is likely to include most genes and an accurate estimate of the copy number for both large and small gene families (Mardis et al., 2002), although information on gene synteny will be missing (Frei et al., 2021). Long reads greatly improve genome assemblies, but centromeric regions, telomeres, and highly repetitive regions can cause problems with any approach in the absence of sufficient sequencing and computational resources. Currently, the two main options for long‐read sequencing are Oxford Nanopore Technologies (ONT or Nanopore) and Pacific Biosciences (PacBio) (Pucker et al., 2022).

Previous studies have compared different DNA extraction methods to obtain sequencing data for a variety of plant lineages (e.g., Abdel‐Latif and Osman, 2017; Pipan et al., 2018; Scobeyeva et al., 2018), although many of these were not intended to generate extractions optimized for long‐read sequencing technology, which requires extremely pure, high‐quality DNA. In the past several years, new protocols have been developed for generating high‐molecular‐weight DNA specifically for long‐read sequencing (e.g., Mayjonade et al., 2016; Li et al., 2020; Jones et al., 2021; Maghini et al., 2021; Russo et al., 2021; Kang et al., 2023; Xie et al., 2023); however, most protocols do not test how each modification may impact sequencing results, and not all methods work on plant lineages with complicating factors such as secondary metabolites, succulence, high fiber content, or other structural considerations. A recent study by Russo et al. (2022) compared the results of two extraction protocols for long‐read sequencing across multiple lineages of plants, yet the impacts of some modifications were still not explicitly shown.

The recommended sequencing depth to generate a relatively complete genome from long‐read data varies depending on the type of sequencing, with 30–50× coverage recommended for Nanopore (Li and Harkess, 2018; Wang et al., 2021a) vs. 15–25× coverage for PacBio HiFi (Wenger et al., 2019; Hon et al., 2020). While the total sequencing output needed for a nearly complete genome assembly has been explored for a variety of methods, the impact of the read N50 (value for which half of the sequencing reads are a given length or longer) for an effective assembly has not been tested to the same extent. The accuracy of the sequencing reads is an important factor as well, and while newer versions of the Nanopore library kits have increased read accuracy, their impact on downstream genome assemblies is still unclear (Sanderson et al., 2022).

Genome sizes across the land plants vary up to 2400‐fold (Soltis et al., 2003), with the primary factor for size increases being the expansion of repetitive elements (Kelly et al., 2015). Indeed, most plant genomes have a similar composition of predicted coding genes (Michael and Jackson, 2013). In a survey of genome sizes across the land plants, Wicker et al. (2017) found the mean haploid size to be 4.7 Gbp; however, the mean haploid size of plants for which a genome has been sequenced is 1.21 Gbp (Kress et al., 2022). This disconnect suggests that even though studies have investigated the evolution of genome size, most plants with large genomes are yet to be sequenced. An understanding of the evolution of genome size and other features of genome composition cannot be complete until the available genomic resources fully reflect the diversity of plant genomes. The Liliales, for example, are one of a handful of taxa within the angiosperms that have evolved giant genomes (Pellicer et al., 2014), while also having the greatest spread in 1C values, ranging from approximately 1.5 to 147 Gbp (Leitch and Leitch, 2013). While hybridization and polyploidy (i.e., allopolyploidy) are rampant across the angiosperms and often responsible for changes in genome size (Baack et al., 2005; Leitch et al., 2008), the available data do not appear to support polyploidy as the driver of large genomes in the Liliales, with a lack of paleopolyploid events in the ancestral nodes of the order (see Appendix S7 in Landis et al., 2018) compared with the Poaceae, in which a tighter association between genome size and ploidy exists (Heslop‐Harrison, 2012). Despite these notable features and their importance as ornamentals, the genomic resources available for the Liliales are quite sparse (Kress et al., 2022). Publicly available genomic data include a draft genome of *Calochortus venustus* Douglas ex Benth. (Hernández et al., 2022), transcriptomes from eight taxa included in the One Thousand Plant Transcriptomes Project (1KP; Leebens‐Mack et al., 2019), and transcriptomes associated with tuber formation in *Bomarea* Mirb. (Tribble et al., 2021). Using long‐read sequencing technology to sequence the more repeat‐rich genomes of the Liliales and other taxa with giant genomes will help provide the necessary data to better investigate the tempo and mode of genome size evolution.

The focus of this study is the genus *Calochortus* Pursh (Liliaceae; Liliales), a genus of approximately 67 species displaying large variation of floral forms (Patterson and Givnish, 2004). Some species of the genus display an extraordinary range of intraspecific flower color, such as *C. venustus* (Hernández et al., 2022). Floral syndromes of the group include mariposas, cat's ears, star tulips, and fairy lanterns (Patterson and Givnish, 2004). The large variety of floral forms, however, is not likely associated with pollinator specialization (Dilley et al., 2000). Species relationships within the genus are not fully resolved, with previous classifications relying on cytological data (Beal, 1939, 1941; Beal and Ownbey, 1943) or three plastid genes (Patterson and Givnish, 2004). The estimated genome size of *Calochortus* is between 5.3 Gbp (Kew C‐value database; Pellicer and Leitch, 2020) and 6.2 Gbp (*k*‐mer analysis of *C. venustus* Illumina data; Landis et al., unpublished data).

Here, we present modifications to DNA extraction and post‐extraction cleanup steps that will aid researchers in generating de novo genome assemblies for monocot species, which can be especially useful for researchers who may have limited resources or want to assemble genomes from multiple taxa on a limited budget. The methods shown here were developed for the successful Nanopore sequencing of *Calochortus* species, which possess large genomes that require multiple sequencing runs per species. We show the impact of these modifications on the flow cell output, read N50, and number of reads produced per flow cell for sequencing plant genomes in‐house. Specifically, we address (1) which modifications have a significant impact on sequencing results and (2) whether there is a tradeoff between read size and total sequencing output. Four species of *Calochortus* were sequenced with five flow cells per species, except for *C. umbellatus* A. Nelson, for which we were only able to sequence four flow cells due to limitations in the amount of available tissue. This study is meant to help guide those interested in using third‐generation sequencing technology to assemble the genomes of non‐model species, specifically those in the Liliales or other monocot lineages, and help determine which modifications are the most important for generating high‐quality DNA that will improve genome assemblies.

## METHODS

### Sample collection

**Figure 1:**
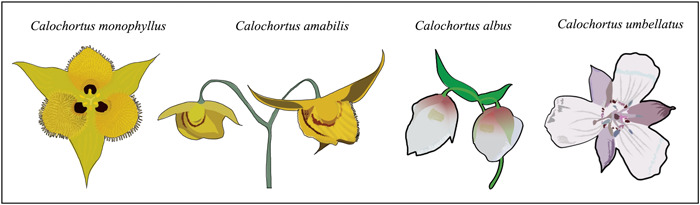
Floral drawings depicting the morphology and pigmentation of the four *Calochortus* species used in this study: *C. monophyllus*, *C. amabalis*, *C. albus*, and *C. umbellatus. Calochortus monophyllus* and *C. umbellatus* possess the star tulip morphology, while *C. albus* and *C. amabilis* display the fairy lantern morphology.

Fresh vegetative and floral tissue of *C. albus* (Benth.) Douglas ex Benth., *C. amabilis* Purdy, *C. monophyllus* Lem., and *C. umbellatus* (Figure 1) were collected in California, USA, and shipped to Cornell University (Ithaca, New York, USA) for sequencing. Freshly collected material was wrapped in damp paper towels, shipped overnight, and stored at −80°C until used. Vouchers of specimens from the same populations were deposited in the University of Wisconsin–Madison Wisconsin State Herbarium (*C. albus* vouchers v0405931WIS and v0405932WIS, *C. amabilis* vouchers v0405929WIS and v0405930WIS, and *C. monophyllus* vouchers v0405937WIS and v0405938WIS; vouchers were not made for *C. umbellatus*). Only one individual of each species was used for sequencing to reduce the impact of heterozygosity, which can be fairly substantial in members of this genus (Hernández et al., 2022). Due to the small stature and ephemeral nature of individuals of the *Calochortus* species used here, the entire collection from a single individual was used to generate sequencing data including flower and leaf tissue.

### Tissue disruption

**Figure 2:**
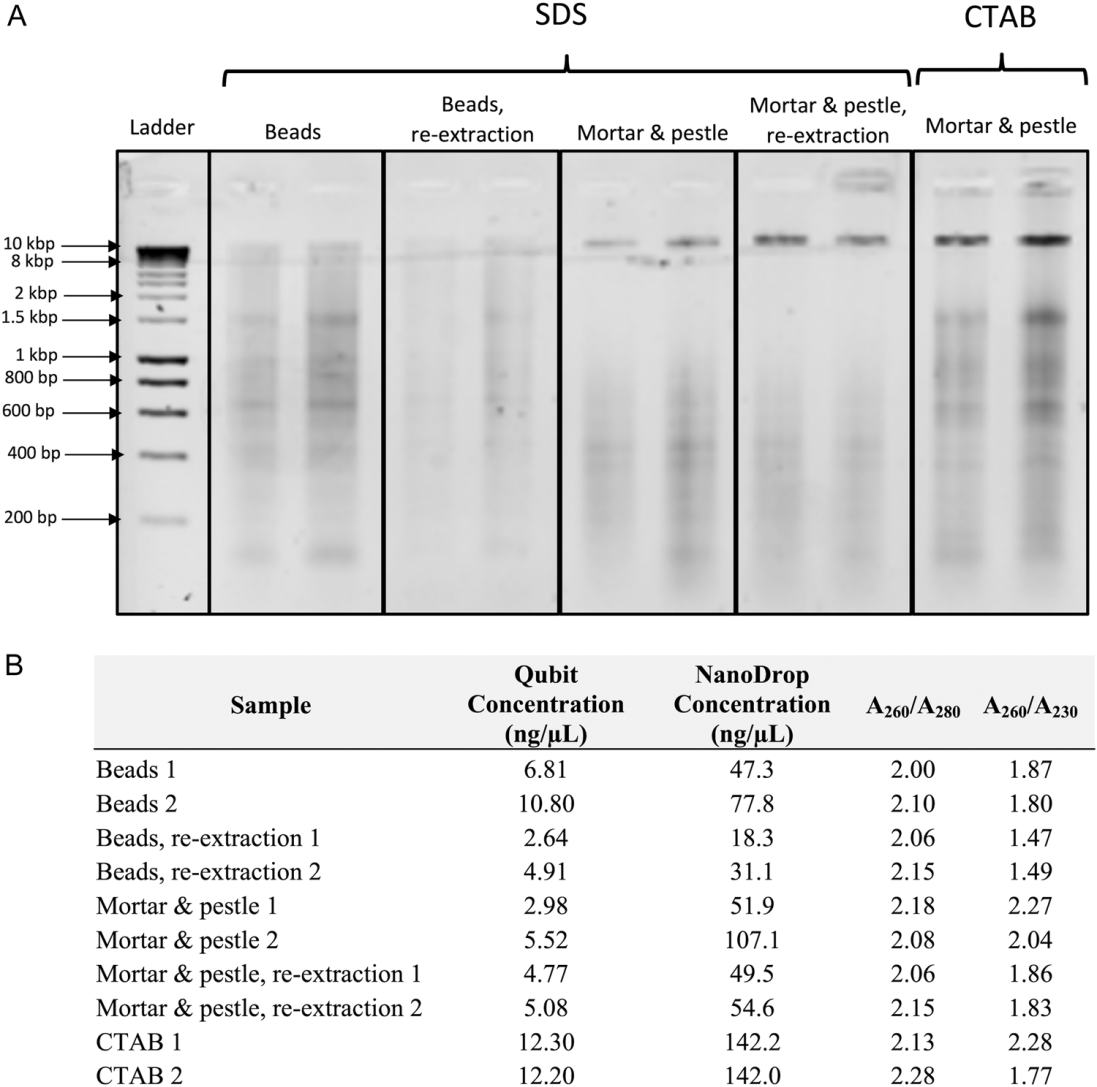
Analysis of DNA extracted using various sample‐grinding techniques and extraction buffers. (A) Comparison of DNA quality produced using different extraction methods, including first and second extractions with beads and SDS, first and second extractions with mortar and pestle and SDS, and extractions with mortar and pestle and CTAB, as visualized on a 1.2% agarose gel. A HyperLadder 1 kb is shown for the estimation of DNA fragment sizes. (B) DNA quantity and quality measurements determined using a Qubit and NanoDrop.

Generating high‐molecular‐weight DNA is important for successful Nanopore sequencing. This starts with grinding the tissue to obtain high‐quality and high‐quantity genetic material (Yockteng et al., 2013; Russo et al., 2022). Previous work in the lab has shown that long‐read sequencing using DNA isolated with a tissue homogenizer results in shorter Nanopore reads (e.g., smaller read N50) than when using a mortar and pestle (Landis et al., unpublished data). As a test case, a single set of extractions was performed to compare the DNA quality between a tissue homogenizer and a mortar and pestle using an optimized sodium dodecyl sulfate (SDS) extraction protocol (Figure 2).

When using a mortar and pestle, it is important to keep the tissue as cold as possible to avoid DNA degradation. If the tissue thaws (indicated by a darkening in color and softened texture), the tissue will not grind finely, which will compromise DNA yield and quality. To maintain cold temperatures and prevent thawing, we first chilled the mortar by filling it with liquid nitrogen. Once fully evaporated, we refilled the mortar and placed plant tissue in the bottom, submerging the tissue. The tissue was ground to a fine powder with a chilled pestle. With a metal spatula, we transferred a volume of approximately 100 µL of ground tissue into individual 1.5‐mL Eppendorf tubes. Next, 700 µL of SDS buffer or 500 µL of cetyltrimethylammonium bromide (CTAB) buffer was added to the ground tissue depending on downstream protocols (see below).

For comparison, we used a Bead Ruptor Elite Bead Mill Homogenizer (OMNI International, Kennesaw, Georgia, USA) to grind the frozen leaf tissue. We added approximately 1 cm^2^ of frozen tissue to tubes each containing two zinc‐plated 0.177‐caliber BBs (Daisy Outdoor Products, Rogers, Arkansas, USA). The tubes were secured in the homogenizer and shaken at a speed of 5.5 m/s for 30 s. To the ground tissue we added 700 µL of SDS buffer and proceeded with the recommended protocol.

### DNA extraction

Two DNA extraction protocols were used for isolating DNA from the ground leaf tissue: a modified CTAB protocol (Doyle and Doyle, 1987) and a modified SDS protocol (Edwards et al., 1991; Konieczny and Ausubel, 1993). Due to previous success in the lab using SDS for high‐quality DNA extractions from monocot samples, CTAB was only used in the initial comparison (Figure 2); all other extractions relied on the SDS approach. When using SDS, the same tissue can be subjected to multiple rounds of extraction by adding fresh buffer and incubating at 65°C; however, the cleanliness of the extracted DNA decreases with each round of extraction, as measured using a NanoDrop 2000 spectrophotometer (Thermo Fisher Scientific, Waltham, Massachusetts, USA) for the ratio of absorbance at 260 nm and 280 nm (A_260_/A_280_) and at 260 nm and 230 nm (A_260_/A_230_). We therefore limited each sample to two rounds of extractions except when there was limited tissue available (i.e., *C. monophyllus*), in which case we performed a third round of extraction (see Table 1 for details). We modified the original SDS method by including a chloroform–isoamyl alcohol cleanup and additional ethanol washes during the precipitation step. For the final elution of DNA, we added 100 µL of 10 mM Tris to each sample and stored them overnight at 4°C. We also modified the original protocol by using cut pipette tips (regular pipette tips modified with a razor blade to remove 3–5 mm from the tip) or wide‐bore tips (from the manufacturer) for all pipetting steps that involved DNA to limit mechanical shearing, promoting the retention of the largest possible DNA fragments.

**Table 1:** Description of sequencing flow cells including modifications in the DNA extraction and cleanup steps. Outputs from the sequencing run include the total output, read length, and number of reads. Columns with a gray background are protocol modifications.

Species	Library	Re‐extracted for a third round	Cut tips for SDS extraction	Cut tips for cleaning	Chloroform for cleaning	Beads for sample grinding	SRE for cleaning	Qiagen for cleaning	Qiagen cleaning repeated	DNA input (total ng)	Sequencing output (Gbp)	Sequencing N50 (kbp)	Median sequencing read size (kbp)	No. of reads (millions)	No. of pores
*C. monophyllus*	1	—	—	✓	✓	✓	✓	✓	—	1390	16.15	8.90	5.79	2.44	1355
*C. monophyllus*	2	✓	✓	✓	✓	✓	✓	✓	—	1040	15.26	8.12	4.87	2.61	1352
*C. monophyllus*	3	—	—	✓	✓	✓	—	✓	—	1161	6.63	8.66	5.09	1.14	1306
*C. monophyllus*	4	✓	—	✓	✓	✓	✓	✓	—	1460	10.65	4.57	1.02	4.99	1334
*C. monophyllus*	5	✓	—	✓	✓	✓	—	✓	✓	1450	7.60	4.26	0.90	3.99	1314
*C. amabilis*	1	—	—	✓	✓	✓	✓	✓	—	933	22.92	8.70	5.04	3.63	1610
*C. amabilis*	2	✓	✓	✓	✓	✓	✓	✓	—	1035	12.80	10.24	5.45	1.87	1737
*C. amabilis*	3	—	—	✓	✓	✓	✓	✓	✓	1620	29.48	7.00	4.23	5.70	1433
*C. amabilis*	4	—	—	✓	✓	✓	—	✓	✓	2330	12.31	8.90	4.23	2.61	1303
*C. amabilis*	5	—	—	✓	✓	✓	—	✓	—	2420	14.31	10.34	5.40	2.13	1553
*C. albus*	1	—	✓	✓	✓	✓	✓	✓	—	844	12.20	12.30	6.80	1.40	1295
*C. albus*	2	—	✓	✓	✓	✓	✓	✓	—	1052	11.20	10.34	5.96	1.54	1157
*C. albus*	3	✓	✓	✓	✓	✓	✓	✓	—	1105	10.03	10.79	5.75	1.35	1404
*C. albus*	4	—	—	✓	✓	✓	✓	✓	✓	1580	15.16	9.02	4.38	1.35	1495
*C. albus*	5	—	—	✓	✓	✓	—	✓	✓	1450	14.86	8.48	5.48	2.43	1299
*C. umbellatus*	1	—	✓	✓	✓	✓	✓	✓	—	920	8.48	13.70	7.46	0.90	1268
*C. umbellatus*	2	—	✓	✓	✓	✓	✓	✓	—	1195	9.65	10.50	5.91	1.34	1304
*C. umbellatus*	3	✓	✓	✓	✓	✓	✓	✓	—	998	7.44	10.00	5.26	1.98	1252
*C. umbellatus*	4	✓	—	✓	✓	—	✓	✓	—	1007	8.87	8.10	4.98	1.49	1231

*Note*: SDS = sodium dodecyl sulfate; SRE = short read elimination.

### Initial quality check

To assess the quality of the DNA generated using different methods of tissue disruption and extraction, as described above, we compared the degree of fragmentation and DNA purity of the samples. Samples differing in tissue disruption (homogenizing beads vs. mortar and pestle), extraction buffer (SDS vs. CTAB), and initial vs. repeated SDS extractions were loaded into a 1.2% agarose gel alongside the Bioline HyperLadder 1 kb (Meridian Bioscience, Cincinnati, Ohio, USA) and run at 120 V for 35–45 min for a complete separation of bands. The quantity of DNA was checked with a Qubit dsDNA Broad Range (BR) Assay Kit (Thermo Fisher Scientific) to determine the concentration (ng/µL), as well as a NanoDrop to measure the concentration (ng/µL) and quality (A_260_/A_280_ and A_260_/A_230_ ratios) of the samples.

### DNA purification

Following extraction, the DNA was left to rehydrate for at least 18 h. Five to 10 extractions from a single individual were combined in a 2‐mL tube and cleaned with homemade cleaning beads (Rowan et al., 2017). If the total volume of the combined extractions was less than 1000 µL, 10 mM Tris was added to increase the total volume to 1000 µL. Approximately a 1× ratio (960 µL) of beads was added to each tube, which were incubated at room temperature for 10 min with gentle inversion/mixing every 2 min. The samples were then placed on a magnetic rack for 5 min (or until all beads were pulled toward the magnet) and the supernatant was removed, leaving the DNA adhered to the beads. The beads were washed twice with 800 µL of 80% ethanol and incubated at room temperature for 30 s while remaining on the magnet to remove the supernatant after each wash. After the final ethanol wash, the tubes were removed from the magnet and allowed to air dry for 3–5 min to avoid extensive drying and cracking. The beads were then incubated with 62 µL of sterile water for 10 min at room temperature to facilitate the DNA dissociating from the beads. The tubes were placed back on the magnet for ~5 min and the eluted DNA was saved for the next cleaning steps. In some cases, the beads became clumped during the initial magnetic separation cleanup step (after the incubation of the DNA and beads, but prior to the ethanol washes) such that the solution remained cloudy beyond the normal incubation period. In those cases, the tubes were left on the magnetic stand for at least 45 min or until the supernatant could be removed without retaining any of the beads to allow for washing with ethanol. The bead cleanup was not applied to one sample (*C. umbellatus* flow cell number four) for which tissue was running low to avoid substantially reducing the total DNA yield.

### DNA quantification, size selection, and cleanup

After extraction and purification, we again quantified the DNA from each extraction with a Qubit dsDNA BR Assay Kit to determine the total yield (ng) and ensure the quantity was sufficient for further processing. In cases where individual samples did not generate the minimum quantity suitable for short read elimination (SRE), i.e., samples with a concentration less than 25 ng/µL, multiple extractions were combined and concentrated by repeating the bead cleanup steps described above after combining multiple tubes. Size selection was performed using the Short Read Eliminator XS kit (Circulomics, Baltimore, Maryland, USA), following the manufacturer's protocol, to completely remove DNA fragments smaller than 5 kbp and progressively remove those up to 10 kbp. Because recovery following this step can be as low as 60% of the input, the SRE step was skipped for those samples for which we had limited amounts of tissue (see Table 1 for details). We noted that samples with a concentration less than 25 ng/µL recover DNA concentrations that are insufficient for library preparation.

The final cleanup step used the DNeasy PowerClean Pro Cleanup Kit (Qiagen, Germantown, Maryland, USA) following the manufacturer's protocol with the exception of the final elution step, which was modified to boost DNA recovery per sample. Specifically, we repeated the DNA elution step, retaining the MB Spin Column after the protocol's “final” centrifugation, placing the column into a new 2‐mL collection tube and adding 50 µL of Solution EB to the center of the filter membrane. The tube was incubated for 1 min at room temperature and centrifuged at 10,000 × *g* for 1 min, resulting in a second elution. All recovered DNA (both elution steps) was quantified using NanoDrop prior to being combined for library preparation. After performing all of the cleaning steps, we are left with pure DNA (or nearly pure DNA), so the NanoDrop readings should be highly similar (between 1:1 to 2:1) to the Qubit readings as no contaminants remain to absorb light and skew the concentration readings.

### Library preparation and sequencing

All cleaned and quantified DNA samples were used to generate libraries for sequencing using the Oxford Nanopore Ligation Sequencing Kit (SQK‐LSK110; Oxford Nanopore Technologies, Oxford, United Kingdom). As recommended, between 1 and 3 µg of DNA were used as input (see Table 1 for specific amounts). Some modifications were made to the standard library preparation instructions provided by Oxford Nanopore. Specifically, if a single tube of recovered DNA from the Qiagen kit was not concentrated enough to hit the target amount of DNA for a given library, the multiple tubes of DNA were kept separate, with each tube treated for end repair following the recommended volumes in the Nanopore protocol. The incubation times for this step were extended from 5 min in the standard protocol to 30 min, more closely following New England Biolab's recommendations for the NEBNext FFPE DNA Repair and Ultra II End Repair enzymes (New England Biolabs, Ipswich, Massachusetts, USA). The appropriate amount of beads was added to maintain the targeted ratio of beads to DNA (1×). For the final step in the library preparation, a total of 13.5 µL of the DNA library was added to the buffer and beads and loaded onto the flow cell. A step‐by‐step protocol for DNA extraction, processing, and library preparation is included in Appendix S1. The manufacturer's recommended steps are not explicitly stated, but specific deviations from the standard protocols are included.

Sequencing was run for 96 h or until all the pores in the flow cell were exhausted. The resulting fast5 files were basecalled with a GPU instance of the Guppy basecaller v6.0.0+ab7925058 (Oxford Nanopore Technologies) with a minimum quality score of five.

### Comparisons and statistical tests

Summary statistics and read size distribution for each flow cell were generated using NanoPlot v1.32.1 (De Coster et al., 2018). Additionally, the length and average base quality of each read were calculated with the fx2tab function in seqkit v2.3.0 (Shen et al., 2016). Regression analyses were performed to determine whether there was a correlation between the input variables (total nanograms of DNA used for library preparation; number of active pores in the flow cell) and output variables (read N50; total output [Gbp] per flow cell; total number of reads) using base R v4.1.2 (R Core Team, 2021). Additional regressions were also performed to identify any relationships between the N50 read length, total output (Gbp), and total number of reads.

Both the total output (Gbp) and N50 value of a sequencing run are widely used metrics to assess the quality of genome sequences before assembly and polishing (Alhakami et al., 2017). We assessed the effects of using cut pipette tips during DNA extraction and of using SRE during cleanup on the total flow cell output using the R function ‘geom_bar’ to generate comparison graphs depicting the total sequencing output (Gbp) and N50 values for all flow cells. To test whether the implemented modifications had a significant result on sequencing, a Mann–Whitney *U* test was performed in R for the following comparisons: cut tip vs. regular tip and SRE vs. no SRE for both the total output (Gbp) and overall N50 of each flow cell.

## RESULTS

### Comparison of extraction methods

The DNA extracted using mortar and pestle tissue disruption showed larger fragment sizes than the bead‐homogenized samples. The former had dark bands on the gel, the largest of which was 10 kbp, as measured alongside the HyperLadder 1 kb (Figure 2A). For the bead‐ground samples, the larger bands were absent, with the bulk of the DNA fragments estimated between 600 and 1500 bp. The CTAB extractions contained the highest DNA concentrations (Figure 2B); however, the DNA was more fragmented (darker bands between 600 and 1500 bp) than that extracted with SDS and the mortar and pestle method, although it remained more intact than DNA extracted using beads.

The re‐extracted SDS samples were of similar quality to the first round of extractions for samples ground with either the beads or mortar and pestle. The concentration of the recovered DNA was approximately half of the initial yield, while the A_260_/A_280_ and A_260_/A_230_ values were lower but still in an acceptable range of nearly pure DNA. Notably, the second DNA extractions were slightly more pure, with a NanoDrop:Qubit ratio close to 10:1, while the initial extractions were closer to 20:1. Pure DNA with no contaminants will have a ratio close to 2:1 or 1:1, resulting in successful Nanopore runs without clogging the sequencing pores.

### Read distributions and regression analyses

Regression analyses for two different input variables (total concentration of DNA for library preparation and initial number of active pores in the flow cell) were compared to the output variables of total Gbp output, read N50, and total number of reads for all flow cells and for the three different categories of treatments (regular tips and SRE, cut tips and SRE, and regular tips and no SRE) (Appendices S2 and S3). The only comparison that resulted in a significant linear correlation was the number of initial active pores and total Gbp output (Appendix S3). No linear relationships between any other variables were observed when considering the plots containing all the flow cells; however, when observing the three different treatment groupings, a non‐significant linear relationship was detected between the total input DNA and read N50 when using cut tips and SRE (*R* ^2^ = 0.29) and regular tips and no SRE (*R* ^2^ = 0.26), between the DNA input and total output with regular tips and no SRE (*R* ^2^ = 0.38), and number of initial active pores and total DNA output with regular tips and SRE (*R* ^2^ = 0.41). Across the three treatments, we did not observe the same general patterns in terms of flow cell production, which indicates that the different modifications of DNA preparation have a direct impact on sequencing efficacy, although these differences are not consistent across all comparisons.

### Sequencing output: Comparison between treatments

**Figure 3:**
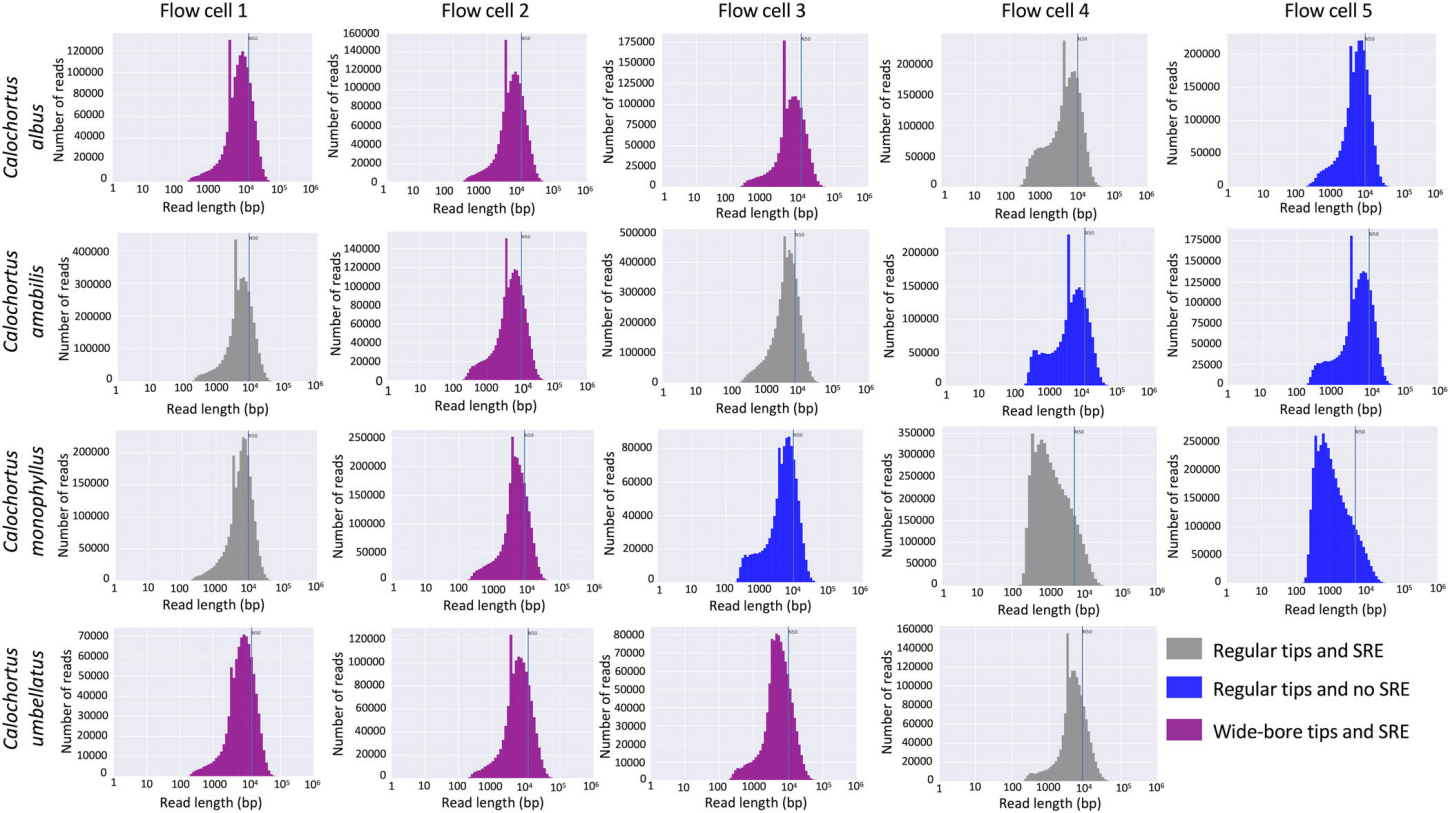
Read distribution for each flow cell on a log scale, highlighting the N50 value. Plots are color coded to represent the different treatments: gray is DNA prepared with regular tips and short read elimination (SRE), purple is DNA prepared with cut or wide‐bore tips and SRE, and blue is DNA prepared with regular tips and no SRE.

**Figure 4:**
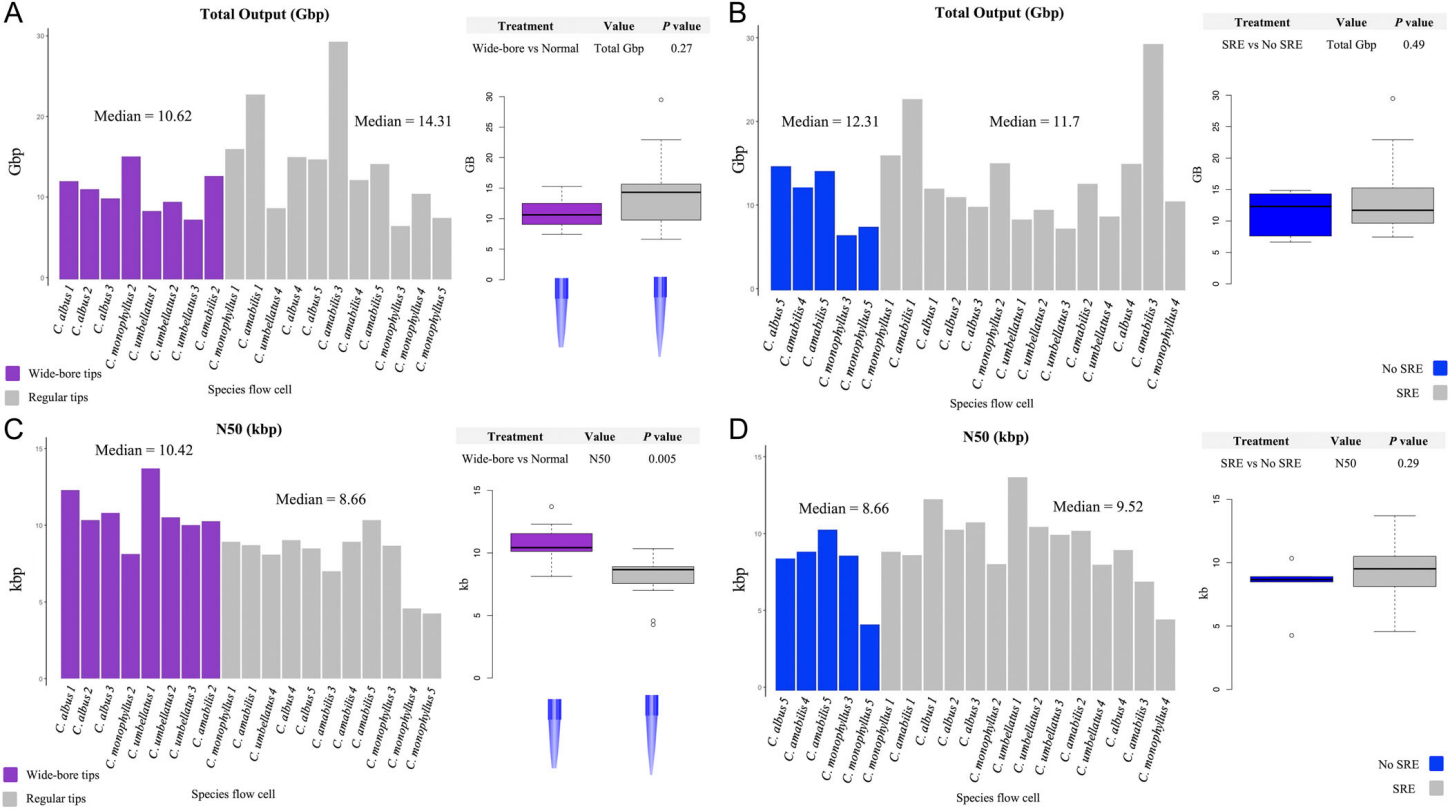
Comparisons of DNA treatments with either the total output (Gbp) per flow cell or read N50 (kbp). (A) Comparison of total output using wide‐bore tips vs. normal tips. (B) Comparison of total output using short read elimination (SRE) vs. no SRE prior to library preparation. (C) Comparison of read N50 using wide‐bore tips vs. normal tips. (D) Comparison of read N50 using SRE vs. no SRE prior to library construction. The *P* values for each plot were determined using a Mann–Whitney *U* test.

The read distributions for each flow cell are shown in Figure 3. All flow cells show a sharp peak around 3000 bp due to a variety of factors, such as use of the long‐fragment buffer (which preferentially retains fragments larger than 3000 bp) during library preparation, incorporation of lambda DNA from the DNA control sample that was used in the library preparation, as well as to the presence of bacterial lineages (e.g., *Escherichia*, *Klebsiella*, and *Staphylococcus*) because the plant material was collected from wild populations. Across all preparation methods, some small sequences (<3000 bp) remain, yet the relative proportion of the short reads to the rest of the reads is smaller in treatments utilizing SRE. The *x*‐axis (read length) is the same across all plots, while the *y*‐axis (number of reads) is drastically different depending on the quality of the sequencing run. We detected a significant difference in N50 values when using cut tips vs. regular tips (*P* = 0.005) (Figure 4). No significant differences were found between the influence of cut tips vs. regular tips on total output (*P* = 0.27), SRE or no SRE on total output (*P* = 0.49), and SRE or no SRE on N50 (*P* = 0.29).

**Figure 5:**
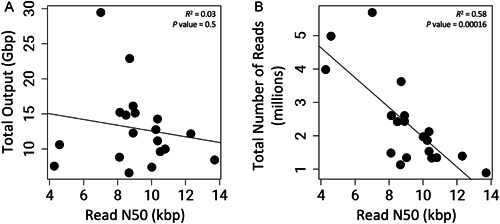
Regression comparing the read N50 to (A) the total output (Gbp) and (B) the total number of reads across all sequencing runs.

Comparing the read N50, total output (Gbp), and total number of reads across all flow cells does reveal tradeoffs; there is a slight negative linear association between read length and total output (*R* ^2^ = 0.03, *P* = 0.5; Figure 5A), with the flow cells producing the largest output having a read N50 between 7 and 9 kbp. Conversely, the relationship between read length and the total number of reads produced in a sequencing run is more pronounced, with longer read lengths producing fewer reads (*R* ^2^ = 0.58, *P* = 0.00016; Figure 5B).

## DISCUSSION

The different sequencing runs of five flow cells across four species of *Calochortus* revealed noticeable differences in DNA quantity and quality when extracted following different preparation methods. Appropriate grinding methods are critically important for generating long reads suitable for genome assembly (Oppert et al., 2019; Penouilh‐Suzette et al., 2020; Zerpa‐Catanho et al., 2021; Russo et al., 2022). While the use of tissue disruptors is important for the high‐throughput processing of many samples for population or phylogenetic sampling studies, these approaches can greatly reduce the amount of high‐molecular‐weight DNA obtained (Figure 2). The settings of our bead‐disruption method were not optimized to produce the most intact DNA possible, so with appropriate tweaking more intact DNA could be recovered; however, the quality of the DNA will still not be as high as when using a mortar and pestle to grind the samples. In this study, we did not carry out Nanopore sequencing on DNA samples extracted with a tissue disruptor or bead beater, but previous sequencing experiments have revealed a drastic reduction in read N50 (4 to 6 kbp) and a marginal reduction in total output (12–18 Gbp) in sequencing members of the Poaceae and their close relatives using this approach (Landis et al., unpublished data).

**Figure 6:**
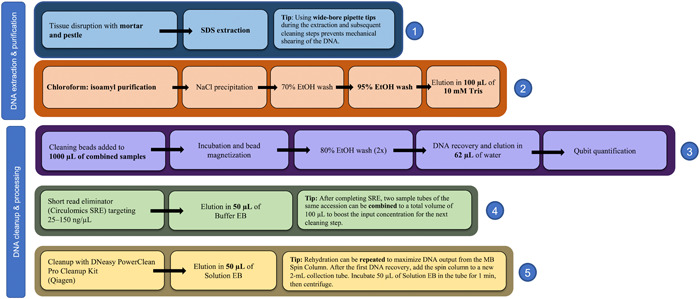
Workflow diagram highlighting modifications to the procedures that impact the sequencing output. Specified modifications for the adapted protocol are shown in bold. Circled numbers indicate the order in which procedures should be followed.

When comparing basic extraction protocols without specific modifications intended for long‐read sequencing, especially in the Liliales, the SDS extraction method appears to provide a better foundation for third‐generation sequencing. Although the CTAB extractions provided more concentrated DNA, the SDS protocol recovered more intact fragments (less smearing observed on a 1.2% agarose gel). For Nanopore sequencing, the quality of the extracted DNA, specifically the cleanliness of the DNA, is arguably more important for a successful sequencing run than its concentration. Dirty DNA will clog the sequencing pores within the first 2–3 h, whereas clean DNA may only result in a small decline in active sequencing pores after 36–48 h. Additionally, SDS methods allow for multiple rounds of extraction to be carried out on the same ground tissue. Each subsequent extraction produces slightly less output (although between the first two extractions this decrease is often minimal; Figure 2) and slightly less pure DNA (as measured by the NanoDrop A_260_/A_280_ and A_260_/A_230_ values). Up to three rounds of extractions can yield suitable DNA for Nanopore sequencing, as observed in the sequencing runs of *C. monophyllus* and *C. umbellatus* (Table 1). In this study, we tested multiple modifications for DNA extraction and processing prior to the library preparation specifically targeted for Nanopore sequencing (Figure 6, Appendix S1). The only modification that had a statistically significant impact on either the total output of a flow cell or the read N50 was the use of cut tips (*P* = 0.0065); cut tips produced a median N50 of 10.42 kbp, while using regular tips produced a median N50 of 8.66 kbp (Figure 4). While statistically nonsignificant, the use of cut tips does appear to produce a smaller output per flow cell compared with regular tips (cut tips median Gbp = 10.62, regular tips median Gbp output = 14.31). In theory, removing small fragments prior to sequencing should drastically improve the sequencing yield (Murigneux et al., 2020; Wang et al., 2021b), especially given that Nanopore preferentially sequences shorter reads; however, we do not see a large difference in median output (SRE = 11.7 Gbp vs. no SRE = 12.31 Gbp) or read N50 (SRE = 9.51 kbp vs. no SRE = 8.66 kbp) when using the Short Read Eliminator XS kit. More noticeable differences may have been detected had we used the regular Short Read Eliminator kit (near‐complete depletion of fragments shorter than 10 kbp and progressive depletion of fragments shorter than 25 kbp) or the Short Read Eliminator XL kit (Circulomics) (near‐complete depletion of fragments shorter than 10 kbp and progressive depletion of fragments shorter than 40 kbp); however, much less DNA is recovered with these larger kits, which can cause problems when tissue is limited, as is the case for many *Calochortus* species due to their minimal vegetative tissue and the production of only one or a few flowers per individual.

Isolating pure DNA is important for producing sequencing runs with a solid output. Secondary compounds that typically inhibit plant DNA extractions in some taxa, including polysaccharides, polyphenols, and phenolic compounds (Friar, 2005; Sahu et al., 2012; Bailey et al., 2022), can easily clog pores in a flow cell and result in poor sequencing runs. One modification we made to the standard SDS protocol was a chloroform–isoamyl alcohol cleanup step, which is standard in most CTAB protocols (Doyle and Doyle, 1987). While this step is not necessary for Illumina sequencing following SDS extractions (Valderrama et al., 2020, 2022), the chloroform cleanup greatly improves the A_260_/A_280_ and A_260_/A_230_ values recorded with the NanoDrop to levels closer to pure DNA (A_260_/A_280_ between 1.8–2.0; A_260_/A_230_ between 2.0–2.2), which are necessary for good Nanopore sequencing runs. The final step in our DNA cleaning process was the Qiagen DNeasy PowerClean Pro Cleanup Kit, which removes inhibitory substances in the extracted DNA, including polysaccharides and lipids. Although this step does fragment the DNA, sometimes by as much as half (N50 of ~20 kbp to ~10 kbp), the total output per flow cell is boosted 2–3×, as was seen in the long‐read sequencing of *Costus* L. (Landis et al., unpublished data; Valderrama et al., 2022). Performing a second elution of the DNA from the spin column allows for a greater recovery of DNA. The product of the second elution is usually at a lower concentration than the first elution, but would otherwise be lost entirely. Recently, Pushkova et al. (2019) showed that hairpin RNA structures can clog the pores of the flow cell; however, with the inclusion of the SRE size selection, most of these structures should be removed based on fragment size.

Optimizing Nanopore sequencing for total output is important when working with midsize to large genomes in the Liliales or other plant groups (Pellicer et al., 2014). For a contiguous genome assembly, a target of 50× coverage is ideal (Li and Harkess, 2018); therefore, multiple Nanopore flow cells are necessary for each species or individual. In the present study, we observed a tradeoff between the length of sequencing reads vs. total output and total number of reads (Figure 5). We found a strong association between the read length N50 and the total number of reads produced, while the association between read length and total output was not significant. In each of the flow cells presented in this study, we see a large peak around 3000 bp (Figure 3). Our BLAST searches against the National Center for Biotechnology Information (NCBI) database revealed that these 3000‐bp fragments are a combination of *Calochortus* genome reads and reads from bacterial lineages infecting the wild‐collected material. While these reads impact our sequencing statistics, they can be identified and excluded during scaffolding without impacting the quality of the final assembly (Li and Harkess, 2018). Having only analyzed 19 sequencing runs, we are likely lacking the power to identify some of the associations between variables (Jenkins and Quintana‐Ascencio, 2020). A similar pattern was observed by Russo et al. (2022), who showed that when the N50 was doubled across PromethION runs, the total output was reduced by at least 25% and the total number of reads was reduced by 76%. Longer reads approaching 50 kbp, referred to as ultra‐long reads, can make the assembly of large complex genomes easier (Frei et al., 2021). Even a relatively small number of ultra‐long reads, approaching a 5× coverage of the genome, can greatly improve the contiguity of genome assemblies (Jain et al., 2018); however, generating longer reads requires more sequencing runs, which might sometimes be cost prohibitive. If the goal is to optimize a sequencing run for the total output or coverage depth of the targeted genome, then reducing the DNA fragment size could be highly beneficial, especially if funding is limited.

There are other factors that can have a large impact on the quality of long‐read sequencing runs that have not been thoroughly tested in this study. One issue largely out of the control of researchers is the quality of the Nanopore flow cell itself. We saw a significant association between the initial active number of pores and total output (Appendix S3). Many factors can contribute to sequencing performance beyond the flow cells themselves; indeed, the sequencing run that produced the largest amount of data in a single run (29.5 Gbp) had a mid‐distribution number of active pores (1433 pores) and read N50 (7 kbp) value. The purity of DNA that goes into library preparation is a major contributor. When quantifying pure DNA prior to library construction, a 1:1 or 2:1 ratio of NanoDrop to Qubit readings is ideal. The age of the tissue (e.g., old vs. new leaves) and tissue type (floral vs. vegetative) can greatly impact the quality of the DNA that is extracted. Developmentally older material may also accumulate secondary compounds, which makes DNA extraction more difficult (Moreira and Oliveira, 2011). In many species, including our own observations in *Calochortus*, cleaner DNA yields can be obtained from flower material than vegetative material (Tamari et al., 2013). Species‐level differences may also exist when undertaking evolutionary genomics studies, especially for distantly related individuals or taxa growing under different environmental conditions. Taxonomic differences are unlikely to be a major factor in this case, given that all four species used are closely related and grow in similar habitats (Patterson and Givnish, 2004).

## CONCLUSIONS

Generating long‐read sequencing for de novo genome assemblies of non‐model taxa is becoming cheaper and easier with improvements in Nanopore sequencing technology; however, preparing DNA for sequencing is still an important step with implications in sequencing quality. Important factors that can determine whether a sequencing run is successful include the cleanliness of the DNA, the degree of DNA fragmentation, and the DNA quantity. Here, we showed that for Nanopore sequencing in the Liliales, grinding the samples with a mortar and pestle followed by DNA extraction using SDS extraction buffer yields the highest‐quality DNA. Utilizing cut tips during the DNA extraction and processing steps generates longer reads, with the tradeoff of fewer sequencing reads and a potential smaller total output. Processing the DNA with the Short Read Eliminator XS kit removes the smallest fragments, although the impact on the overall distribution of read sizes is not consistent. Increasing the purity of the DNA by removing secondary compounds may be more important for generating a greater quantity of sequencing reads. Optimizing sequencing runs for total output to achieve the recommended coverage for a reasonable genome assembly may mean reducing the targeted DNA fragment sizes. This is important when sequencing non‐model taxa on a small budget. The steps shown here are directly applicable to the Liliales, but the same modifications have also been applied to several other lineages of monocots with similar success.

## AUTHOR CONTRIBUTIONS

C.D.S., J.B.L., and S.R.S. conceived the ideas and designed the methodology. G.Y.D., J.B.L., and A.I.H. performed the wet lab work. E.E. collected the material for genomic extractions in the field. F.W.L. and J.Z. provided guidance for wet lab procedures. C.M.T., N.K., P.C., and T.G. facilitated and executed tissue collection in California, USA. J.B.L. and G.Y.D. conducted the analyses and wrote the first draft of the manuscript. All authors helped revise the manuscript and gave final approval for publication.

## Supporting information


**Appendix S1**. Stepwise procedure for DNA extraction, subsequent cleaning steps, and modifications to the standard Nanopore library preparation protocol.

**Appendix S2**. Regression plots comparing total DNA input to total output (Gbp; top row), read N50 (middle row), and total number of reads (bottom row). The first column shows all sequencing flow cells, the second column just the samples that were prepared with regular tips and the Short Read Eliminator XS kit (SRE), the third row shows samples prepared with cut tips and SRE, and the fourth column comprises samples prepared with regular tips and no SRE.

**Appendix S3**. Regression plots comparing the number of initial active pores in each Nanopore flow cell to the total output (Gbp; top row), read N50 (middle row), and total number of reads (bottom row). The first column shows all sequencing flow cells, the second column just the flow cells containing samples that were prepared with regular tips and the Short Read Eliminator XS kit (SRE), the third row shows flow cells with samples prepared with cut tips and SRE, and the fourth column comprises the flow cells containing samples prepared with regular tips and no SRE.

## Data Availability

Data files including summary statistics for each flow cell and inputs for figure generation, as well as scripts for the analyses and plotting, have been uploaded to GitHub (https://github.com/jblandis/Calochortus_genome_sequencing_methods).
