# Supplementary material for: Balancing read length and sequencing depth: Optimizing Nanopore long‐read sequencing for monocots with an emphasis on the Liliales
Source: Appl Plant Sci. 2023 Jun 6;11(3):e11524. doi: 10.1002/aps3.11524 (PMC10278932; doi:10.1002/aps3.11524)
Supplement: Supplementary file 1 — Appendix S1. Stepwise procedure for DNA extraction, subsequent cleaning steps, and modifications to the standard Nanopore library preparation protocol. [file APS3-11-e11524-s002.docx]

**Appendix S1.** Stepwise procedure for DNA extraction, subsequent cleaning steps, and modifications to the standard Nanopore library preparation protocol.

**SDS EXTRACTION**

Adapted from the Hake Lab protocol (UC Berkeley) by Chodon Sass and Clarice Guan. Last modified September 2022 for Nanopore sequencing by Jacob Landis and Gisel De La Cerda.

**Note**: Modifications from the original protocols specified for this lab procedure are **bolded**.

When performing repeat extractions, it is recommended that samples from the first extraction reach step 7 of the protocol, at which point the tubes containing the ground tissue can return to step 4 (with 400 µL of added sodium dodecyl sulfate [SDS] extraction buffer). While re-extraction tubes sit in the hot block, samples containing the first extraction may continue with the protocol until reaching the isopropyl alcohol (IPA) precipitation (step 12). While the first extractions precipitate, wait for the re-extractions to finish incubating on the hot block; then proceed with the protocol until the re-extractions reach IPA precipitation. The first set of extractions may continue with the protocol once the −20°C incubation is complete. If both sets of extractions remain in the freezer overnight, they can be washed and eluted together the next day.

1. Prior to extraction, turn on a heat block or oven and set to 65°C.
2. **Pour liquid nitrogen** **into a mortar** and allow it to completely evaporate. **Refill** the mortar and submerge fresh tissue (frozen −80°C), adding liquid nitrogen as needed to keep everything cold. Using the chilled **mortar and pestle**, grind the tissue into a fine powder.
3. Divide the ground tissue among **24 tubes** (maximum), being careful not to put too much tissue in each tube. Ground tissue may occupy **100–300 µL** in a tube without packing it down.
4. Add 700 μL of SDS extraction buffer to each sample. Incubate at 65°C for an hour, inverting the tubes manually every 15 min to mix the sample tissue with the buffer.
5. Centrifuge the samples for 2 min at a speed of 13,000 × *g*.
6. Using **wide-bore tips**, transfer 400 µL of supernatant from the tubes containing the tissue into new, labeled 1.5-mL sample tubes.

**Note:** Wide-bore tips are commercially available, or they can be created in-house by cutting 3–5 mm from regular tips with a sterile razor blade.

1. Before proceeding to the next step, add 400 µL of SDS extraction buffer to each sample tube containing tissue and place the tubes back on the hot block for the repeat extraction. Let the tubes sit for one hour, inverting the tubes manually every 15 min to mix the tissue sample with the buffer. Proceed with step 5 for the re-extraction.
2. To the newly labeled tubes containing the supernatant, add in **400 µL of 24:1 chloroform:isoamyl alcohol**, invert approximately 10 times to ensure samples are fully mixed, then spin for **10 min** at 13,000 × *g*.
3. With a P1000 pipette set to 400 µL using **wide-bore tips**, transfer the clear supernatant to a new, labeled tube. Be careful to only aspirate the top aqueous layer from the sample.
4. Add 173 μL of 5 M NaCl to each new sample tube. Mix gently by inverting, then spin for 2 min at 13,000 × *g*.
5. Using a P1000 pipette set to 580 μL, use **wide-bore tips** to transfer as much supernatant as possible into newly labeled 1.5-mL sample tubes. Be careful not to disturb the pellet in the tube.
6. Add **400 μL of 100% IPA** to each sample tube. Mix gently by inversion, then leave at −20°C for at least one hour. Samples may be left in the freezer for as long as **overnight**. Longer times in the freezer can boost DNA recovery but at the cost of dirtier DNA. For Nanopore sequencing, one hour is best.
7. Spin samples for 5 min at 13,000 × *g*, then pour the supernatant into a waste bin using one smooth pouring motion. Take care to only invert the tube one time to avoid losing the DNA pellet.
8. Add 500 μL of 70% ethanol (EtOH) to each sample tube, inverting until the pellet is suspended to ensure a thorough wash. Spin tubes for **3 min** at 13,000 × *g*, then pour out the supernatant into the waste bin using one smooth motion.
9. Repeat step 14 with **95% EtOH**. After you pour the supernatant out of each tube, keep the tube upside down and open as you set it on a paper towel.
10. Let the tubes dry for 30–45 min (until no EtOH remains).
11. Add **100 μL** of **10 mM Tris (pH 8.0)** to each sample tube and keep **overnight** at 4°C to allow for full resuspension. Thereafter, samples can be stored at 4°C (short term) or −20°C (long term).

**SDS extraction buffer (makes 50 mL):**

10 mL 1 M Tris (pH 8.0)

2.5 mL 5 M NaCl

2.5 mL 0.5 M EDTA

2.5 mL 10% SDS

32.5 mL H_2_O

It is recommended to aliquot all other reagents into the tube **before** adding water, so that one only needs to fill the tube up to the 50-mL mark rather than measure 32.5 mL in a separate tube to be poured.

**CLEANING BEADS**

Adapted from Rowan et al. (2017). Last modified September 2022 for Nanopore sequencing by Jacob Landis and Gisel De La Cerda.

**Note:** Before using cleaning beads, remove them from 4°C storage to allow equilibration to room temperature (**about 20 min**).

1. **Combine** five to 10 samples of one accession/individual into a 2-mL tube. If the total volume is less than 1000 µL, **add** **10 mM Tris** to reach 1000 µL.
2. Add **960 µL** (roughly 1× ratio) of cleaning beads so as to not overfill the tube.
3. Incubate the sample at room temperature for **10 min** to allow the DNA fragments to adhere to the beads. Carefully mix the sample by inversion every 2 min.
4. Transfer the tube to an appropriately sized magnetic rack and incubate for 5 min until all beads are pulled toward the magnet and the solution is clear.

**Note:** In this step, the beads may clump such that the solution remains cloudy beyond the normal incubation period. Keep the tube on the magnetic stand for at least **45 min** or until the supernatant can be removed and transferred to a separate tube without retaining any of the beads.

1. Remove and discard the supernatant while the tubes are sitting on the magnetic rack. Be careful not to disrupt the bead pellet.
2. Add **800 µL** (or the volume necessary to completely cover the beads) of freshly prepared 80% EtOH. Wait 30 s, then remove the ethanol without disrupting the beads. Repeat this wash.
3. After removing the second ethanol wash, allow the beads to dry at room temperature until all the ethanol has evaporated (**1–5 min** at most to avoid cracking the pellet).
4. Remove the tube from the magnet and add **64 µL** of sterile water. Gently flick the tube to ensure beads are resuspended and thoroughly mixed with the water.
5. Incubate the tube at room temperature for **10 min** to allow the DNA to come into solution.
6. Transfer the tube to the magnetic rack and incubate for about 5 min or until all the beads are pulled toward the magnet and the water appears clear.
7. With a **wide-bore tip**, transfer **62 µL** of the eluted DNA to a new tube and proceed with DNA quantification, size selection, and cleanup.

**SIZE SELECTION**

For the size selection, the Circulomics Short Read Eliminator XS kit (Circulomics, Baltimore, Maryland, USA) manufacturer’s protocol was followed with the exception of the final elution steps (steps 9 and 10), which were specified to maximize the DNA concentration per sample while ensuring complete elution.

9. After completing the second ethanol wash of the sample, add **50 µL** of Buffer EB (provided in the SRE kit) to the tube.

10. Incubate at **4°C overnight** to allow the full rehydration of the DNA.

**Note:** After the Short Read Eliminator XS steps are complete and if samples are available, two tubes of the same accession can be combined to a total volume of **100 µL** to boost the input concentration for the following cleaning step.

**CLEANUP**

For the cleanup, the DNeasy PowerClean Pro Cleanup Kit (Qiagen, Germantown, Maryland, USA) manufacturer’s protocol was followed with the exception of the final elution step (step 15), which was modified to boost the DNA recovery per sample.

15. After the elution step in the standard protocol, **retain** the MB Spin Column and place it into a new 2-mL collection tube.

16. Add **50 µL** of Solution EB (provided in kit) to the center of the filter membrane and incubate at room temperature for 1 min.

17. **Centrifuge** at 10,000 × *g* for 1 min at room temperature.

18. Discard the MB Spin Column. The two sets of tubes for each sample can be combined for the library preparation.

**LIBRARY PREPARATION**

All cleaned and quantified DNA samples were used to generate libraries for sequencing using the Oxford Nanopore Genomic DNA by Ligation SQK-LSK110 kit (Oxford Nanopore Technologies, Oxford, United Kingdom) with the following modifications of the standard protocol. The same modifications are applicable to all recent versions of the ligation kit, including the newest version SQK-LSK114.

**End repair**

In a 0.2-mL thin-walled PCR tube, mix the following:

1 μL DNA CS (control strand, provided in kit)

**48 µL of DNA (25–50 ng/µL) cleaned with the PowerClean Pro Cleanup kit**

3.5 μL NEBNext FFPE DNA Repair Buffer (New England Biolabs, Ipswich, Massachusetts, USA)

2 μL NEBNext FFPE DNA Repair Mix (New England Biolabs)

3.5 μL NEBNext Ultra II End-prep reaction buffer (New England Biolabs)

3 μL NEBNext Ultra II End-prep enzyme mix (New England Biolabs)

Mix gently by flicking the tube, then spin down briefly.

Using a thermal cycler, incubate at 20°C for **30 min** and 65°C for **30 min**.

**Bead cleanup**

After transferring the end repair product to a new Eppendorf tube, add 60 µL of resuspended homemade cleaning beads or AMPure beads (Beckman Coulter, Brea, California, USA) to the end-prep reaction and mix.

Spin down the sample and pellet on a magnet until the eluate is clear and colorless (**2 min**). Keep the tube on the magnet and pipette off the supernatant.

Proceed with the ethanol wash as stated in the protocol.

**Adapter ligation and cleanup**

Proceed with adapter ligation as specified in the standard protocol. After the incubation, add in 40 µL of resuspended cleaning/AMPure beads and incubate for 5 min.

Spin down the sample and pellet on a magnet for **2 min**. Keep the tube on the magnet, and pipette off the supernatant.

Wash the beads by adding either **200 μL of** Long Fragment Buffer or **200 µL** of Short Fragment Buffer. Flick the beads to resuspend, spin down, and then return the tube to the magnetic rack for **3 min** to allow the beads to pellet. Remove the supernatant using a pipette and discard.

Remove the tube from the magnetic rack and resuspend the pellet in 15 μL of Buffer EB. Spin down and incubate for 10 min at 37°C to improve the recovery of long fragments.

Pellet the beads on a magnet for **2 min** until the eluate is clear and colorless.

Pipette **13.5 µL** directly into the loading mixture, and save the remaining sample for Qubit quantification (optional).

**Library loading**

In a new tube, prepare the library for loading as follows:

37.5 μL Sequencing Buffer II (SBII)

25.5 μL Loading Beads II (LBII), mixed immediately before use, or Loading Solution (LS) if using

**13.5 μL** DNA library

After priming the flow cell with 800 μL of flush buffer and waiting 5 min, lift the covering of the SpotON sample port on the flow cell.

Load **300 μL** of the priming mix into the flow cell via the priming port (not the SpotON sample port), avoiding the introduction of air bubbles.

Add **75 μL** of sample (library, loading buffer, and loading beads) to the SpotON sample port in dropwise fashion.

**REFERENCES**

Rowan, B. A., D. K. Seymour, E. Chae, D. S. Lundberg, and D. Weigel. 2017. Methods for genotyping-by-sequencing. *In* S. White and S. Cantsilieris [eds.], Genotyping, 221–242. Methods in Molecular Biology, vol. 1492. Humana Press, New York, New York, USA.
