# Supplementary material for: Balancing read length and sequencing depth: Optimizing Nanopore long‐read sequencing for monocots with an emphasis on the Liliales
Source: Appl Plant Sci. 2023 Jun 6;11(3):e11524. doi: 10.1002/aps3.11524 (PMC10278932; doi:10.1002/aps3.11524)

DOI 10.1002/aps3.11524

APPENDIX S3. Regression plots comparing the number of initial active pores in each Nanopore flow cell to the total output (Gbp; top row), read N50 (middle row), and total number of reads (bottom row). The first column shows all sequencing flow cells, the second column just the flow cells containing samples that were prepared with regular tips and the Short Read Eliminator XS kit (SRE), the third row shows flow cells with samples prepared with cut tips and SRE, and the fourth column comprises the flow cells containing samples prepared with regular tips and no SRE.

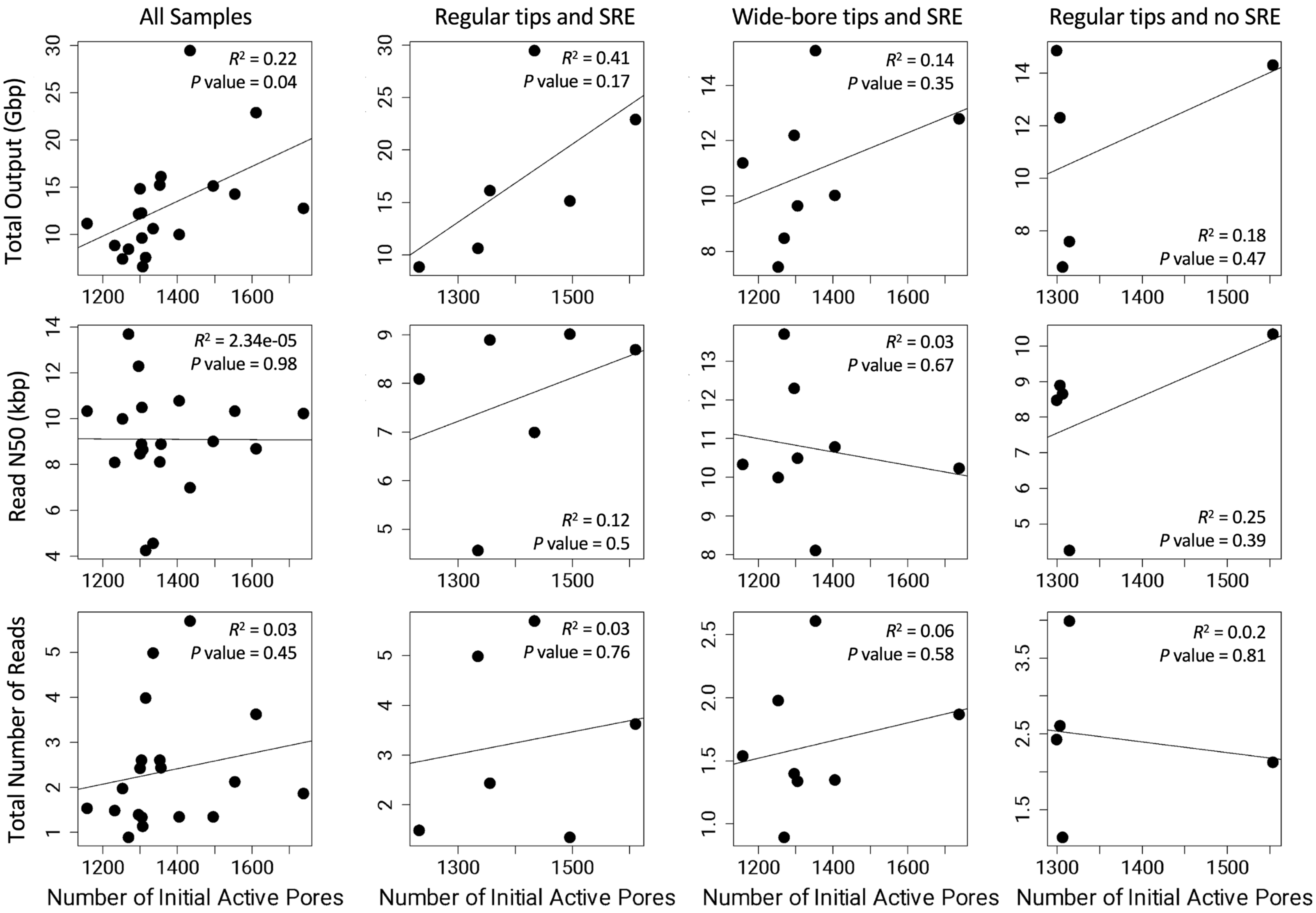

Supplement: Supplementary file 3 — Appendix S3. Regression plots comparing the number of initial active pores in each Nanopore flow cell to the total output (Gbp; top row), read N50 (middle row), and total number of reads (bottom row). The first column shows all sequencing flow cells, the second column just the flow cells containing samples that were prepared with regular tips and the Short Read Eliminator XS kit (SRE), the third row shows flow cells with samples prepared with cut tips and SRE, and the fourth column comprises the flow cells containing samples prepared with regular tips and no SRE. [file APS3-11-e11524-s003.pdf]
